# Acute Limb Ischemia—Much More Than Just a Lack of Oxygen

**DOI:** 10.3390/ijms19020374

**Published:** 2018-01-26

**Authors:** Florian Simon, Alexander Oberhuber, Nikolaos Floros, Albert Busch, Markus Udo Wagenhäuser, Hubert Schelzig, Mansur Duran

**Affiliations:** 1 Department of Vascular and Endovascular Surgery, Heinrich-Heine-University Medical Center Düsseldorf, 40225 Düsseldorf, Germany; alexander.oberhuber@med.uni-duesseldorf.de (A.O.); nikolaos.floros@med.uni-duesseldorf.de (N.F.); wagmar@stanford.edu (M.U.W.); Hubert.schelzig@med.uni-duesseldorf.de (H.S.); mansur.duran@med.uni-duesseldorf.de (M.D.); 2 Department for Vascular and Endovascular Surgery, Klinikum Rechts der Isar Technical University, 81675 Munich, Germany; albert.busch@mri.tum.de; 3 Division of Cardiovascular Medicine, Stanford University School of Medicine, Stanford, CA 94305, USA

**Keywords:** pathophysiology, ischemia, reperfusion, hypoxia, free radicals

## Abstract

Acute ischemia of an extremity occurs in several stages, a lack of oxygen being the primary contributor of the event. Although underlying patho-mechanisms are similar, it is important to determine whether it is an acute or chronic event. Healthy tissue does not contain enlarged collaterals, which are formed in chronically malperfused tissue and can maintain a minimum supply despite occlusion. The underlying processes for enhanced collateral blood flow are sprouting vessels from pre-existing vessels (via angiogenesis) and a lumen extension of arterioles (via arteriogenesis). While disturbed flow patterns with associated local low shear stress upregulate angiogenesis promoting genes, elevated shear stress may trigger arteriogenesis due to increased blood volume. In case of an acute ischemia, especially during the reperfusion phase, fluid transfer occurs into the tissue while the vascular bed is simultaneously reduced and no longer reacts to vaso-relaxing factors such as nitric oxide. This process results in an exacerbative cycle, in which increased peripheral resistance leads to an additional lack of oxygen. This whole process is accompanied by an inundation of inflammatory cells, which amplify the inflammatory response by cytokine release. However, an extremity is an individual-specific composition of different tissues, so these processes may vary dramatically between patients. The image is more uniform when broken down to the single cell stage. Because each cell is dependent on energy produced from aerobic respiration, an event of acute hypoxia can be a life-threatening situation. Aerobic processes responsible for yielding adenosine triphosphate (ATP), such as the electron transport chain and oxidative phosphorylation in the mitochondria, suffer first, thus disrupting the integrity of cellular respiration. One consequence of this is irreparable damage of the cell membrane due to an imbalance of electrolytes. The eventual increase in net fluid influx associated with a decrease in intracellular pH is considered an end-stage event. Due to the lack of ATP, individual cell organelles can no longer sustain their activity, thus initiating the cascade pathways of apoptosis via the release of cytokines such as the BCL2 associated X protein (BAX). As ischemia may lead to direct necrosis, inflammatory processes are further aggravated. In the case of reperfusion, the flow of nascent oxygen will cause additional damage to the cell, further initiating apoptosis in additional surrounding cells. In particular, free oxygen radicals are formed, causing severe damage to cell membranes and desoxyribonucleic acid (DNA). However, the increased tissue stress caused by this process may be transient, as radical scavengers may attenuate the damage. Taking the above into final consideration, it is clearly elucidated that acute ischemia and subsequent reperfusion is a process that leads to acute tissue damage combined with end-organ loss of function, a condition that is difficult to counteract.

## 1. Introduction and Background

Although the underlying patho-mechanisms are similar, acute and chronic ischemia differ in regards to the age groups predominantly affected (deatils further below). Peripheral arterial occlusive disease (PAOD) affects more than 202 million people worldwide, a slow process that increasingly narrows the arterial lumen and reduces blood flow, resulting from atherosclerotic changes in the vascular walls [1]. In contrast, acute limb ischemia is typically either due to one of two pathogenic processes. In the elderly (>65 years), up to 85% of acute lumen occlusions form as arterial thrombosis at pre-damaged atherosclerotic sites, a chronic process which suddenly causes a severe and acute medical emergency. Non-atherosclerotic peripheral embolism accounts for 10% to 15% of acute ischemia, which may affect all age groups [2]. In the latter process, an arterial embolism from the heart or another proximal source of diseased large vessels, such as aneurysms with intraluminal thrombus formations, may travel to extremities, thus causing limb ischemia. This is primarily observed in patients suffering from heart or vessel diseases such as arrhythmia, aortic aneurysm, and dissection [3,4,5,6,7]. As mentioned above, acute local thrombotic occlusions deriving from chronic atherosclerotic plaques mostly cause prolonged acute ischemia. In such an event, the body engages in compensatory mechanisms to divert blood flow through peripheral collateral vessels formed over time, ensuring a minimum level of oxygen supply. This chronic etiopathology causes a broad range of symptoms over time, while patients are constantly at risk to develop a severe peripheral ischemia [8]. Aside from these processes, there are also rare diseases, such as paradox embolism or phlegmasia coerulea dolens, in which acute arterial ischemia occurs even when the origin primarily stems from the venous system [9].

There are several underlying reasons as to why an atherosclerotic plaque that develops over years suddenly destabilizes and leads to an acute thrombotic occlusion. These reasons are effectively examined in histological compositions. During the growth of an atherosclerotic plaque, smooth muscle cells are stimulated by a cytokine gradient to migrate from the media into the intima, producing major components of the extracellular matrix and forming a fibrous cap. This cap stabilizes the plaque and the necrotic core [10]. Macrophages that are located under this cap digest accumulated oxidized low-density lipoprotein (LDL), amplifying inflammatory reactions as they release cytokines and metalloproteinases. The combination of a thin fibrous cap with a strongly necrotic decaying core containing sprouting capillaries into the hypoxic plaque causes micro-bleedings and further recruits inflammatory cells. The gradual reduction of smooth muscle cells causes a destabilization of the plaque and finally leads to rupture of the fibrous cap with subsequent thrombosis and occlusion of the vessel [11,12]. Whether the plaque ruptures during physical work or without additional stress depends on the precise composition of its structure. Given a large number of inflammatory cells primarily settling in the plaque shoulder regions, the fibrous cap is likely to rupture at these sites, even at rest. This is due to the accelerated degradation of structural proteins causing destabilization of the plaque edges. In contrast, a rupture in the middle of the atheroma is primarily caused by calcification, as this reduces elasticity of the fibrous cap. Hence, during additional mechanical stress such as physical exertion, a sudden plaque rupture is at risk. Although both processes might be pathophysiological different, thrombus formation is commonly shared [13,14].

Chronically under-oxygenated tissue forms collateral networks, which become more augmented in size and area as the rate of vasculature narrowing decreases. In particular, when the collateral network density is higher, the area of tissue affected by hypoxic conditions is smaller. These collaterals are formed based on two parallel processes: firstly by angiogenesis, i.e., the sprouting of small vessels from existing arterioles, and secondly by arteriogenesis, i.e., the enlargement of the lumen of pre-existing arterioles. However, arteriogenesis contributes more to tissue blood supply [15,16]. That being said, the clinical picture of acute occlusion differs broadly among patients with pre-existing damaged tissue; elderly people are more likely to have collaterals as a residual blood reserve than younger patients, who do not form biological bypasses, as their acute ischemia is not plaque-related [17].

While oxygen is a necessary requirement for the creation and sustenance of life, it can also cause serious damage, as seen in ischemia and reperfusion. Irrespective of the various pathophysiological processes underlying ischemia, the end-stage remains the same: a sudden lack of oxygen in the tissue, accumulation of metabolites, production of reactive oxygen species (ROS) and an inflammatory reaction in conjunction with tissue swelling. Acute ischemia of the extremities can lead to consequential damage of varying severity with a loss of limb tissue and function. The loss of light touch sensation, accompanied by severe pain, followed by skin numbness and paralysis of the musculature are indicative of irreversible tissue loss [16]. Therefore, a sudden interruption of blood supply to a limb is a medical emergency that threatens with the possibility of limb loss and even death. Clinical symptoms of an acute limb ischemia are provided in Table 1 described by Pratt as the 6 Ps [18].

## 2. Collaterals

Healthy tissue does not contain enlarged collaterals, which are formed in chronically under-oxygenated tissue. In the case of an acute arterial occlusion of an extremity, it is crucial to distinguish between healthy tissue without collaterals and pre-damaged tissue with biological bypasses, since collaterals may ensure a minimal oxygen supply of the extremity despite the occlusion. As mentioned above, the underlying mechanisms for the formation of this enhanced collateral blood flow are sprouting vessels from pre-existing vessels (via angiogenesis) and lumen extension of the arterioles (via arteriogenesis) [15]. While disturbed flow patterns with associated local low shear stress upregulate angiogenesis-promoting genes, elevated shear stress may trigger arteriogenesis due to increased blood volume [19]. Specifically, the formation of collateral vessels is stimulated by hypoxic conditions intermittently caused in bouts of ambulation. During ambulation, stenoses in conduit arterial vessels do not permit the body from fulfilling its demands for oxygen supply. As a result, this acts as a stimulus for the formation of collateral vessels. This exercise-induced formation of collateral networks is further enhanced by the release of growth factors from mechanically stressed tissue to improve blood delivery [17,20].

Angiogenesis is a complex process that can be further divided into several sub-processes that can run simultaneously: Sprouting angiogenesis is characterized by the proliferation of endothelial cells forming the structural foundation of new capillaries from pre-existing vessels. Secondly, the enlargement of capillaries that should be distinguished from angiogenesis discussed below and lastly the splitting of vessels into smaller ones, called intussusceptive angiogenesis [21,22,23]. As briefly mentioned earlier, a driving force in angiogenesis is the undersupply of oxygen to tissue, which results in the release of different growth factors such as FGF (fibroblast growth factor), VEGF (vascular endothelial growth factor), and HIF (hypoxia inducible factor) [24,25]. HIF-1α plays an important role in this context as a barometric marker for measuring relative O_2_ pressure (pO_2_) in the tissue. In the event of lowered pO_2_, HIF-1α mRNA is upregulated and, besides increasing erythropoiesis and glycolysis by erythopoietin, induces the transcription of the vascular endothelial growth factor (*VEGF*) gene [26].

The basal membrane and surrounding tissue are macerated by proteases. A rudimentary extracellular matrix (ECM) is created as the permeability for plasma proteins increases in the tunica intima. Next, endothelial cells and pericytes proliferate and migrate by lateral movement into this preliminary matrix. The accumulation of further cells and alignment of the cell groups along a gradient of ephrin and semaphorin (partly membrane-bound and partly secreted proteins) result in a capillary sprout, continuously increasing in diameter and length and ultimately forming a lumen [27]. After a stabilization phase, the activation of protease inhibitors such as TIMP (tissue inhibitors of metalloproteinases) and PAI-1 (plasminogen activator inhibitor-1) leads to the formation of new basal membranes and cell-to-cell contacts [23]. Despite the formation of new capillaries, the prevention of an acute ischemia is not possible, since flow resistance in the smallest vessels is several times higher than in the conduit vessels. Only the tissue surrounding the capillary can be supplied by angiogenesis, as a much higher demand of capillaries may be required to prevent complete tissue ischemia [28].

**Figure 1:**
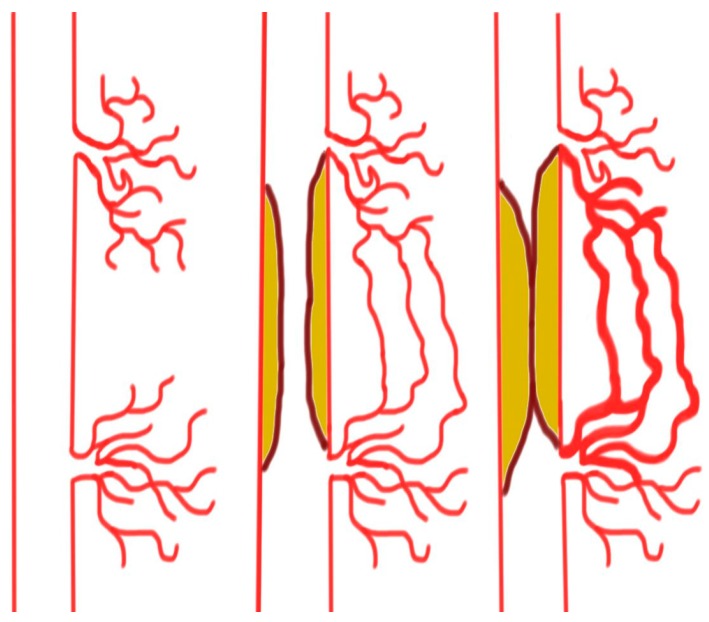
Slow progressive narrowing of a conduit vessel increases the shear forces and blood volume, resulting in collateral formation.

Arteriogenesis is an effective method of forming biological bypasses and is characterized by the lumen expansion of preexisting vessels. This enlargement of arterioles accommodates for the transport of blood volume several magnitudes greater than those of unenlarged arterioles [29,30]. Under healthy conditions, these vessels play only a marginal role for sufficient blood supply to major extremities. However, if gradually progressive atherosclerotic narrowing of a conduit vessel occurs, blood flow is increasingly diverted into these arterioles. This increased supply of blood volume increases the shear forces on the glycocalyx receptors of the endothelium, activating calcium ion channels and stimulating phospholipase activity. In response, the production of cyclic adenosine monophosphate (cAMP) and the release of prostaglandins enhance phosphatidylinositol-4,5-biphosphate 3-kinase (PI3K) activity and subsequently trigger endothelial nitric oxide synthase (eNOS) phosphorylation. The resultant production of nitric oxide leads to smooth muscle cell relaxation and ultimately lumen expansion [31,32]. Furthermore, with lumen expansion, the pressure on the vessel walls forces the arterioles to become permeable to plasma proteins [33]. The incipient reaction is very similar to the above-mentioned mechanism of angiogenesis meaning that arteriogenesis employs the expression of metalloproteinases (MMPs), which decompose the basal membranes and collagen fibers and soften the tissue, thus providing new space. This process is further enhanced by recruited inflammatory cells that displace surrounding cells. However, the recruited inflammatory cells also release cytokines such as MCP-1 (monocytes chemoattractant protein 1) that stimulate migration and proliferation of smooth muscle cells. The resultant cluster organization and re-consolidation of these new cell groups allows the vessel to withstand increased pressure levels and simultaneously increase the diameter of the arterioles by up to 20 times [34]. An overview of arteriogenesis is provided in Figure 1. Changes in the number and organization of endothelial cells and fibroblasts also contribute to vascular remodeling. The pulsatile pressure on the vessel wall is an appropriate stimuli for not only an increase in the arteriole diameter but also in cell mass [35,36]. The increase in diameter and length of arterioles associated with arteriogenesis causes substantial distortions, often referred to as corkscrew collaterals on angiograms. However, these morphological distortions do not prevent a sufficient delivery of blood over long distances to hypoxic tissues, thus ensuring a minimum level of oxygen supply.

## 3. Edema

In the case of an acute ischemia, and especially during the reperfusion phase, malfunctions in membrane integrity and membrane potential lead to altered intracellular electrolyte levels. Intracellular acidosis and cellular degeneration occur. The venous pH-value continuously falls and equilibrates to the intracellular pH-value level after approximately 2 h [37]. Fluid transfer into the tissue is caused by disrupted cellular homeostasis in hypoxic cells. Albumin enters the interstitium and accelerates the formation of tissue edema due to its hydrophilic properties. In this manner, edema contributes to impaired perfusion and reduction of oxygen exchange due to the extended diffusion distances in the tissue. Depending on the extent of perfusion reduction, the edema can be severe and lead to a further deterioration of blood flow via compression of the arteries, further causing a loss of volume into the tissue [38]. While the vascular bed is reduced by increasing pressure, it loses sensitivity to vaso-relaxing factors such as nitric oxide. This results in a cyclic process furthered by increased peripheral resistance leading to additional lack of oxygen. The most affected tissue is the musculature that atrophies following the loss of myosin [39]. When pressure in the muscle increases, the capillaries collapse, the tissue becomes pale, and muscle cells and neurons both suffer irreversible damage from cell death 4–6 h after the onset of ischemia [40]. The re-establishment of sufficient arterial blood flow during reperfusion aggravates edema in the musculature and causes further swelling in pre-defined compartments. This condition, in which the pressure within the fascia lodge exceeds systolic blood pressure levels, is known as compartment syndrome and leads to irreversible ischemic damage of the tissue, contributing to a renewed deterioration of blood circulation [41].

LBF = (PA − PV)/R

Local blood flow (LBF) is characterized by the difference of arterial pressure (PA) and the venous pressure (PV) divided by vascular resistance (R).

Due to the swelling of the musculature, arterial vessels are compressed and react to this additional mechanical irritation with a pronounced vasospasm. In such a situation, surgical pressure release via the incision of the fascia may relieve the tissue pressure and drain the edema [42]. In order to protect blood vessels from thrombosis, occlusions, and dangerous intraluminal pressure levels, a number of antithrombotic factors is expressed by the healthy endothelium. These factors include prostacyclin, nitric oxide, thrombomodulin, and plasminogen activators. Furthermore, because the glycocalyx of the luminal side of the endothelium is highly negatively charged, negatively charged platelets are repelled from the vessel wall. The specific degree of negative charge on glycocalyces varies among vessels, thus regulating which molecules can pass through the barrier and enter interstitial space. However, this barrier can be damaged by ischemia and reperfusion, impairing the ability to protect the tissue against severe fluid influx. Cell-to-cell connections, formed by occludins, adhesion molecules, cadherins, and gap-junctions, along with integrin receptors such as fibronectin, also play a vital role in regulating the fluid influx into the tissue [43,44]. When maximum tissue tolerance to occlusion is exceeded, thrombus formation is increased, and subsequent shedding of the gycocalyx exacerbates leukocyte and platelet adhesion, in turn further increasing fluid influx and thrombus formation [45,46]. The same processes can occur during reperfusion. Since this coagulation preferably takes place in small vessels, immediate removal of the occlusion can neither restore perfusion to the oxygen-consuming tissue nor completely eliminate the edema [47].

## 4. Inflammation

The ischemic and reperfusion (I/R) process is accompanied by the recruitment of inflammatory cells, which enhance inflammation through the release of various cytokines. Many inflammatory cytokines, such as IL-1, IL-6, TNF-α, and thromboxane B2, are released into systemic circulation. Concomitantly, other inflammatory responses, such as the adhesion and migration of leukocytes from the endothelium into underlying tissue, occur in the venules. Once initiated, systemic inflammation is aggravated by complementary cascades, which can be activated through three pathways: the classical pathway via activation of the C1-complex, the alternative pathway via spontaneous C3 hydrolysis, and the lectin pathway via pathogen binding by mannose-binding lectin (MBL). In the classical pathway, circulating IgM binds to C1q on the surface of ischemic cells, leading to the cleavage of C3 into C3a and C3b by C3 convertase. The deposition of C3b eventually leads to the formation of the C5b-9 membrane attack complex (MAC), causing cell destruction by perforation of the cell membrane. In conjunction with this degradation, recruited macrophages release prostaglandin E2, and neutrophils release interleukins and leukotrienes. The activated cleaved complement proteins then adhere to the endothelium of the capillaries and serve as chemoattractants for recruitment of additional leukocytes. In addition, reactive oxygen species (ROS) that damage cell membranes by lipid peroxidation enhance inflammation through further neutrophil activation [48,49]. These activated neutrophils then release three major types of granules which serve different functions: primary granules, also known as azurophilic granules, which contain myeloperoxidase (MPO), elastase, and proteases, secondary granules, also known as specific granules, which contain collagenase and apolactoferrin, and tertiary granules, which contain gelatinase. Some components of these granules are directly involved in furthering a self-perpetuating complement cascade activation circuit [50,51,52]. Neutrophils also contain other types of granules, such as secretory vesicles, which release proteins that are capable of damaging the surrounding cells during an inflammatory response [53].

ROS particularly plays an important role during the early reperfusion period, as their levels are even aggravated by the recruitment and subsequent activation of leukocytes [54]. The production of radicals and the migration of local mast cells and macrophages into the surrounding tissue further enhance the inflammatory response to the I/R [55]. However, free radicals not only execute their damaging potential at the intracellular stage, but also immensely disturb the balance between ROS and major physiological scavengers such as nitric oxide (NO). In the presence of ROS, freely available NO is limited. This causes disruption of the regulation of vascular tone, since NO is a major vasodilator produced by the endothelium. Under normal conditions, the production of NO exceeds the formation of radicals, protecting the cell from severe damage and thus adequately regulating the vascular tone to prevent thrombosis. In ischemically damaged arterioles, however, the endothelial-mediated relaxation no longer reacts to NO. The result is a constriction of the vessels and a substantial reduction of oxygen supply to the tissue [56].

## 5. Molecular Mechanisms

The molecular processes underlying ischemia are more clearly visible when broken down to the single cell stage. Because each cell is dependent on energy produced from aerobic respiration, an event of acute hypoxia can be a life-threatening situation. Under ischemic conditions, xanthine dehydrogenase localized in the endothelial cells of skeletal muscle vessels is converted to xanthine oxidase utilizing the electron carrier nicotinamide-adenine dinucleotide (NAD^+^), which is reduced to nicotinamide adenine dinucleotide hydrogen (NADH) in turn [57]. Deposited xanthine oxidase produces highly oxidizing species such as superoxide anion (O_2_^−^) and hydrogen peroxide (H_2_O_2_), which is catalyzed by the myeloperoxidase (MPO) in conjunction with the oxidation of chloride ions to hypochlorous acid (HOCl). These in turn increase membrane permeability and produce more ROS [58,59]. Although HOCl is a non-radical oxidant, it can react with H_2_O_2_ to produce singlet oxygen ^1^O_2_, causing lipid peroxidation in membranes and subsequent membrane leaking [60]. When oxygen is consumed by ongoing metabolism in cells, new oxygen radicals can only be produced during reperfusion when new molecular oxygen is provided by the bloodstream [61]. HOCl can also react with superoxide and ferrous iron (Fe^2+^) to form hydroxyl radicals, which also cause lipid peroxidation and DNA damage [59,62]. Another oxidase, the nicotinamide adenine dinucleotide phosphate (NADPH) oxidase, is also a major source of ROS in endothelial cells, causing, together with accumulating lactate and H^+^, further damage to the endothelium and leading to tissue necrosis [63,64]. Aerobic processes responsible for yielding ATP, such as the electron transport chain and oxidative phosphorylation in the mitochondria, suffer first, thus disrupting the integrity of cellular respiration. As a consequence, cells with high-energy consumption such as neurons are rendered vulnerable. This causes irreversible damage, which manifests in severe pain and the loss of light touch sensation. In addition, damage to the cell membrane results in an imbalance of electrolytes in the cytoplasm, such as intracellular potassium and calcium levels, causing increased inflow of fluid and a consequent decrease in intracellular pH.

Other damaging effects may include cell death induced by defective mitochondrial permeability transition pores (mPTPs). These pores are non-specific and permeable to molecules smaller than 1.5 kDa. They allow entry into the inner mitochondrial membrane during calcium overload, the depletion of ATP, the elevation of phosphate levels, or oxidative stress, all of which appear during ischemia and reperfusion [65]. While they may not have specialized functions in healthy mitochondria, they may be responsible for cell death in the case of severe cell damage [66]. In the context of severely damaged cells, the open mPTPs allow protons to pass through the inner membrane, causing a dissipation of the H^+^ gradient. This results in reduced ATP production by oxidative phosphorylation, and in turn, malfunction of the energy-dependent ion channels, endangering metabolic homeostasis and promoting cell death through activation of phospholipases, nucleases, and proteases [67,68,69].

**Figure 2:**
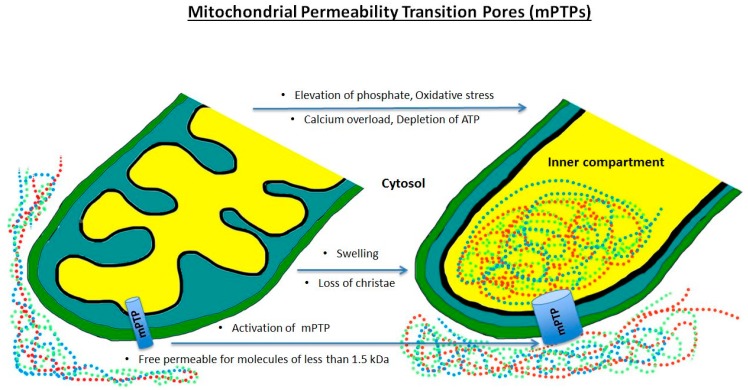
Activation of mitochondrial permeability transition pores resulting in swelling of the inner compartment and loss of membrane function.

In the setting of programmed cell death, commonly known as apoptosis, mPTPs play a role in the rupture of the mitochondria from increased colloid osmotic pressure. The opening of mPTPs causes a colloid osmotic pressure between the inner compartment of the mitochondrium and the cytosol so that fluid will pass inside the open mPTPs (Figure 2). This swelling can be compensated to a certain extent by the folding of the inner membrane. However, when excess pressure is exerted on the outer membrane, the mitochondria ruptures, causing an efflux of mitochondrial components such as cytochrome C and apoptosis inducing factor (AIF). AIF, in combination with cyclophilin A, a matrix protein, then penetrates the cell nucleus and causes DNA fragmentation. In addition, AIF interacts with the simultaneously released endonuclease G, activating another caspase-independent apoptosis pathway. Due to the lack of ATP, cell organelles can no longer sustain their activity and initiate apoptosis of the cell by the release of cytokines such as BAX. Instead, cytochrome C, together with proteins apoptotic protease activating factor 1 (APAF-1), procaspase-9, and dATP, forms a complex called an apoptosome, which triggers apoptosis by activating caspase-3. This process is further accelerated by the release of the second derived mitochondria activator of caspase/direct IAP binding protein with low pl (Smac/DIABLO) and the mitochondrial serine protease high temperature requirement protein A2 (Omi/HtrA2), as they both block caspase-3 inhibitors [68,69,70,71].

**Figure 3:**
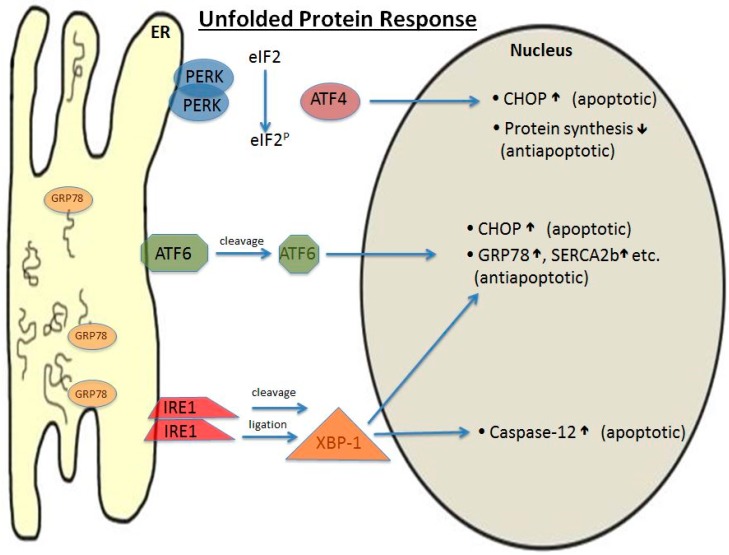
Unfolded protein response initiated by stimuli such as ATP depletion, loss of calcium, and free radicals. ER: endoplasmic reticulum; PERK: protein kinase RNA-like endoplasmic reticulum kinase; GRP 78: glucose regulated protein 78; ATF6: activating transcription factor 6; IRE1: inositol-requiring enzyme 1; XBP-1: X-box binding protein 1; eIF2: eukaryotic initiation factor 2; CHOP: C/EBP homology protein; SERCA2b: sarcoplasmic/endoplasmic reticulum calcium ATPase 2b; ↑: increase; ↓: decrease.

Another vulnerable organelle is the endoplasmic reticulum (ER), which performs in its lumen post-translational modifications of the amino acid strands during protein biosynthesis. This process can come to a halt during ischemic damage caused by, e.g., disturbance of Ca^2+^ concentration, oxidative radicals, and the loss of ATP, which result in mis-modulated proteins that no longer function properly. The result is an accumulation of nonfunctional proteins, as well as stress-related proteins. While stress-related proteins serve to have a protective effect on the cell, they are also capable of further damaging the cell [72,73,74]. Therefore, it is important for the cell to cope with this situation so that either protein biosynthesis is suspended until the cell has recovered or is completely abolished in an act of apoptosis to reduce damage to the organism. This so-called unfolded protein response (UPR) affects the cell in several ways (Figure 3).

First, the glucose regulated protein 78 (GRP78) dissociates from ER-transmembrane effector proteins like the protein kinase RNA-like endoplasmic reticulum kinase (PERK), inositol-requiring enzyme 1 (IRE1), and the activating transcription factor 6 (ATF6) in large numbers and binds the “unfolded proteins” located in the ER lumen to keep the proteins in line for folding. In the case of a heavily misfolded protein, GRP78 initiates the ubiquitination of the protein, labeling it for degradation in the proteasome [73]. This process may lead to the accumulation of misfolded proteins in the ER during ischemia, suppressing protein synthesis by reducing tRNA levels. In parallel, transcription factors are activated to modulate downstream signaling of target genes involved in protein biosynthesis [75]. Thus, the recovery process to regain full ER functionality is contingent on the availability of GRP78. In more detail, dissociated GRP78 induces translocation of ATF6 to the golgi membrane, where it is further processed and subsequently translocates to the nucleus to upregulate transcription of more GRP78, as well as apoptotic proteins [76]. The second transmembrane protein, IRE1, which is activated by the dissociation of GRP78, produces, via several mRNA-splicing and ligation steps, the active product XBP-1, which has overlapping antiapoptotic effects with ATF6 in the nucleus but is also capable of inducing pro-apoptotic Caspase-12 [77,78].

Another component of the UPR is the C/EBP homology protein (CHOP), a cytosolic protein that is expressed in healthy cells only at low levels. In the case of ER stress, it is upregulated and may either induce apoptosis or promote cell survival. CHOP accumulates in the nucleus where it regulates the downstream signaling of certain target genes. In particular, the phosphorylation of the eukaryotic initiation factor 2 alpha (eIF2α) by the kinase PERK inhibits protein biosynthesis and activates transcription factor ATF4 and CHOP, which in turn produces both pro- and anti-apoptotic effects. While, at first sight, this expression of both pro- and anti-apoptotic factors seems to be counterproductive, the net ratio between these factors ultimately dictates which pathway the cell may undergo.

## 6. Systemic Reactions

During I/R, metabolites of the affected extremity are released into circulation so that non-ischemic organs such as the kidney and lungs become involved in inflammation, even though they are not primarily affected [79,80]. Ultimately, an unrestrained post-ischemic reaction can even lead to multi-organ failure and death. This is due to exhaustion caused by continuous energy-consuming processes in the cells. When there is an acute interruption of blood flow, the tissues of these organs limit their energy consumption, switching from aerobic to anaerobic metabolism. Nevertheless, ischemic organs of the extremities, such as the musculature, remain the most affected. Of the molecules that are abundantly released by ischemic muscle tissue, creatinine phosphokinase, myoglobin, lactate dehydrogenase, and, most importantly, potassium interfere with the entire organism and contribute to inflammatory responses. As mentioned earlier, inflammation occurs in the entire organism and initiates a generalized activation of the complement cascade. Activated components of this cascade bind to the endothelium of the capillaries and lead via chemotaxis to an additional accumulation of leukocytes. These processes are associated with an increased production of oxygen radicals [48].

However, not only mesenchymal organs but also the vessels themselves react to systemic inflammation by disrupting the auto-regulation of vasodilatation and vasoconstriction. The result is a vasospasm that strongly contracts the peripheral arterial vessels and represents an excessive vasoconstrictorial reaction of a vessel to a stimulus. The increase of hydrostatic pressure when changing from a supine to an upright standing position may be as high as 100 mmHg, which is why the body physiologically reacts with vasoconstriction in resistance vessels. This vasoconstriction reduces capillary perfusion by up to 70% and limits capillary leakage, thus preventing tissue edema [81]. In the case of acute hypoxia, this regulatory system is disturbed, which leads to an inadequate end-organ oxygen supply.

Some organs are more frequently affected than others. A severe complication during reperfusion is crush kidney. The pathophysiology of crush kidney starts with rhabdomyolysis resulting from muscle degradation along with consecutive leakage of, e.g., myoglobin and creatine phosphokinase. Excess levels of plasma myoglobin lead to occlusion of arterioles in the glomerula of the kidney. The result is acute renal failure indicated by the absence of urine production. This effect is further exacerbated by hypovolemia and aciduria [79,82]. Another highly vulnerable mesenchymal organ is the lungs, which respond to systematic inflammation with increased vascular permeability, thus causing pulmonary edema. Functionally, this is indicated by impaired gas exchange and reduced lung compliance. During recruitment of neutrophils as part of the general inflammatory response, activation of the complement cascade results in the release of major inflammatory cytokines such as TNF-α, leukocyte adhesion molecules (LAMs), and P-selectin into the circulation. In the process, basal membranes and other membrane proteins can be destroyed, resulting in respiratory stress syndrome (ARDS) and alveolar edema. Thus, severe damage to the interstitial tissue critically impairs lung function [80,83,84,85,86].

Apart from the kidney and lungs, the heart should be considered as a potentially involved organ for a number of reasons. During the reperfusion of previously ischemic muscle tissue, plasma hyperkalemia results from the excess potassium released from destroyed muscle cells. A well-established complication resulting from this is arrhythmia. Techniques that have been used to limit this adverse effect of reperfusion include hyperventilation, membrane stabilization with calcium or magnesium, ion buffers, and i.v. glucose in combination with insulin [87]. The same phenomenon accounts for elevated lactate levels, which may cause reduced contractility of the heart. Other effects include acidosis, which interferes with the effects of catecholamines and cytokines such as TNF-α, thus decreasing both diastolic and systolic heart function [88,89,90]. Ultimately, each organ can be affected by the systemic reaction following the local I/R of an extremity.

## 7. Treatment Options

The treatment of acute limb ischemia is based upon the Rutherford classification, a clinical staging system for describing the severity of peripheral ischemia (Table 2) [8]. While patients in Class I may suffer from minor symptoms, their severity and number increases in higher classes. According to this classification, end-stage patients (Class III) need immediate revascularization and major amputations may ultimately be considered. There are numerous surgical and interventional approaches to treat acute peripheral ischemia, and most recent advances have further improved patient outcomes.

The Fogarty thrombembolectomy maneuver is a well-established, feasible, and safe surgical approach that is widely performed. Nevertheless, the complexity of this procedure depends on the approach site and the localization of the occlusion. To improve patient outcomes, the catheter-based thrombectomy can be combined with an intraoperative angiography to confirm complete clot removal. Given these substantial advances, modern guidewire catheter techniques (so called hybrid procedures) may promote the reliability of secure and complete clot removal to further reduce the morbidity associated with acute arterial occlusions as indicated by recent small-scale clinical trials [91,92]. However, the paradigm shift from open surgical towards endovascular therapies does not come without catheter-specific complications. In this regard, the physician may not rely on sufficient backflow after the thrombembolectomy maneuver as a valid indicator for complete blood removal as the peripheral vessel wall is fragile and machano-sensitive which can induce a significant vasospasm [93]. Besides extensions of the traditional Fogarty thrombembolectomy maneuver technique, the latest pharmacological advances are encouraging. As complete clot removal is challenging in small vessels, the intra-arterial injection of thrombolytic agents has been proven beneficial [94].

In the absence of major contraindications (e.g., gastrointestinal bleeding), the catheter-directed thrombolysis (CDT) seems promising for the removal of a freshly formed thrombus. Embedding the tip of a multiple sidehole infusion catheter in the thrombus ensures an even distribution of the thrombolytic agent. Once the tip has been placed correctly, the agent can be administrated either continuously or in a pulse/spray infusion. According to a systematic meta-analysis, no application modus was found to be favorable [95]. Nevertheless, administrating the thrombolytic agent into the thrombus accelerates thrombolysis considerably when compared to more proximal catheter tip placement.

Large lumen catheters are utilized to perform a manual aspiration thrombectomy (MAT). An effective MAT uses dynamic catheter withdrawal maintaining continuous negative pressure to aspirate the thrombus using a 50 mL syringe. This procedure can be combined with local thrombolysis. Despite a high technical success rate and a considerable cost-effectiveness, MAT has not become widely used [96,97].

Several different techniques are summarized under the term “mechanical thrombectomy.” For instance, a fast-spinning head driven by a helix rotating inside the catheter is used for “mechanical thrombus fragmentation.” The fragments can be aspirated by ports located behind the catheter head. The technique delivers high technical success and low complication rates, but additional local thrombolysis may be necessary in specific cases [98,99].

Another mechanical thrombectomy is the so-called “rheolytic thrombectomy.” The method uses a double-lumen device to sequentially fragment and aspirate the thrombus. A saline solution can be administrated in combination with a thrombolytic agent through the first lumen, causing the fragmentation of the clot. Thereafter, the second lumen induces a negative pressure of up to 600 mmHg, enabling the aspiration of the liquidized clot debris. Several studies have reported apparently successful outcomes when applying this method in different clinical settings such as thrombosis, thrombosed stents, and occluded surgical bypasses [100,101,102,103].

Despite general issues with interventional applications, there are method-specific complications. In particular, the high velocity jets can mechanically destroy erythrocytes. The hemolysis may cause severe side effects such as pancreatitis, renal insufficiency, hypotension, and arrhythmia [104,105,106]. A technically more advanced device combines the rheolytic thrombectomy with a non-manual aspiration thrombectomy catheter. Combining these two catheters allows an adequate blood circulation immediately after recanalization, thus not causing hemolysis. That being said, the method is feasible for patients with contraindications such as hepatic failure.

In summary, these techniques demonstrate that recent advances have caused a paradigm shift towards endovascular therapies for peripheral ischemia. Endovascular techniques provide new therapeutic options for patients with multimorbidity, and predicted methodological improvements in the nearer future will likely expand their application range. Despite promising reports within the last decade, large-scale prospective studies are still pending to evidently prove the effectiveness of these therapies.

## 8. Conclusions

Acute ischemia of an extremity is a vascular emergency characterized by pain, swelling and loss of function of the limb, and consecutive tissue necrosis and apoptosis. The degree of tissue damage is contingent on the outreach of the collateral network. Severe tissue destruction do not only manifest in consequences for the affected limb, but also in effects endangering the whole body. Considering that parts of the underlying patho-mechanisms have previously been published over the last century, it can be understood that this disease involves a convoluted network of regulatory mechanisms, many of which are particularly difficult to mitigate. Innovations and methodological advances have recently caused a paradigm shift towards less invasive endovascular therapies in the treatment of acute ischemia.

## Figures and Tables

**Table 1:** Clinical symptoms of acute limb ischemia according to Pratt.

6 Ps	Symptom	Explanation
1.	Paleness	Paleness of the Skin
2.	Pain	Aggravating ischemic pain
3.	Paresthesia	Ascending paresthesia of the skin
4.	Pulselessness	Absence of pulse
5.	Paralysis	Ascending paralysis of the muscles
6.	Prostration	Fatigue, shock, agitation

**Table 2:** Rutherford classification of acute limb ischemia [8].

Class	Category	Prognosis	Sensory Loss	Muscle Weakness	Arterial Doppler	Venous Doppler
I	Viable	No immediate limb threat	None	None	Audible	Audible
IIA	Threatening:marginal	Salvageable if treated promptly	Minimal–none	None	Inaudible	Audible
IIB	Threatening:immediate	Salvageable if treated immediately	More than just toes	Mild–moderate	Inaudible	Audible
III	Irreversible	Limb loss or permanent damage	Profound	Profound	Inaudible	Inaudible
